# Economic Impacts on Human Health Resulting from the Use of Mercury in the Illegal Gold Mining in the Brazilian Amazon: A Methodological Assessment

**DOI:** 10.3390/ijerph182211869

**Published:** 2021-11-12

**Authors:** Leonardo Barcellos de Bakker, Pedro Gasparinetti, Júlia Mello de Queiroz, Ana Claudia Santiago de Vasconcellos

**Affiliations:** 1 Leonardo B. Bakker Assessoria, São Clemente Street, Rio de Janeiro 254, Rio de Janeiro 22260-004, Brazil; 2 Conservation Strategy Fund, Av. Churchill 129, Rio de Janeiro 20020-050, Brazil; pedro@conservation-strategy.org; 3 Julia Queiroz Consultoria Desenvolvimento Verde, Maria Angelica Street, Rio de Janeiro 382, Rio de Janeiro 22461-152, Brazil; julia.melloqueiroz@gmail.com; 4 Laboratory of Professional Education in Health Surveillance, Joaquim Venâncio Polytechnic School of Health, Oswaldo Cruz Foundation, Rio de Janeiro 21040-900, Brazil; ana.vasconcellos@fiocruz.br

**Keywords:** mercury, methylmercury, artisanal small-scale gold mining, Amazon, human health, economic valuation

## Abstract

Artisanal small-scale gold mining (ASGM) in the Amazon results in the dumping of tons of mercury into the environment annually. Despite consensus on the impacts of mercury on human health, there are still unknowns regarding: (i) the extent to which mercury from ASGM can be dispersed in the environment until it becomes toxic to humans; and (ii) the economic value of losses caused by contamination becomes evident. The main objective of this study is to propose a methodology to evaluate the impacts of ASGM on human health in different contexts in the Brazilian Amazon. We connect several points in the literature based on hypotheses regarding mercury dispersion in water, its transformation into methylmercury, and absorption by fish and humans. This methodology can be used as a tool to estimate the extent of environmental damage caused by artisanal gold mining, the severity of damage to the health of individuals contaminated by mercury and, consequently, can contribute to the application of fines to environmental violators. The consequences of contamination are evaluated by dose-response functions relating to mercury concentrations in hair and the development of the following health outcomes: (i) mild mental retardation, (ii) acute myocardial infarction, and (iii) hypertension. From disability-adjusted life years and statistical life value, we found that the economic losses range from 100,000 to 400,000 USD per kilogram of gold extracted. A case study of the Yanomami indigenous land shows that the impacts of mercury from illegal gold mining in 2020 totaled 69 million USD, which could be used by local authorities to compensate the Yanomami people.

## 1. Introduction

The Minamata disaster in Japan, in which thousands of people were seriously impacted by mercury dumped by various industries, culminated in the Minamata Convention in 2013. As a result, mercury’s use has been restricted [1] and it is now considered by the World Health Organization (WHO) as one of the six most dangerous substances to health due to its high toxicity and the risks it poses to human health and the environment [2].

Mercury is a heavy metal widely distributed across the planet and is therefore classified as a global pollutant [3,4]. This metal can be found in nature in three main chemical forms: ionic mercurial forms (e.g., Hg[II]), in its elemental form (e.g., Hg0), and in organomercurial forms (e.g., methylmercury) [5]. Although all mercurial forms have the potential to cause toxic health effects on people, methylmercury is the most dangerous [6]. This organomercurial species affects the central nervous system, causing neurobehavioral effects, motor coordination disorders, and cardiovascular diseases [7,8,9]. Since it chronically affects the population, its effects can arise over many years and cause severe damage to an entire generation. This mercurial form is especially harmful to pregnant women due to the fact that the fetal brain is more sensitive to the action of methylmercury, causing many neurodevelopment problems to occur including mental retardation, learning delays, visual and auditory alterations, and other harmful effects [10,11,12].

Despite its damage to human health, mercury is still widely used in legal and illegal gold mining in Brazil, an activity that has been growing every year due to the high gold price and lack of inspections [13]. The situation becomes even more serious since this increase is largely concentrated in indigenous areas, mainly affecting the Yanomami and Munduruku traditional territories [14,15]. Gold mining uses mercury during the amalgamation process, which unites mercury with gold. Although much of the mercury is reused in the process, some is lost and is dispersed in rivers, soils, and the atmosphere [16,17,18,19,20]. The Brazilian Ministry of the Environment estimated that, in 2016, between 18.5 and 221 tons of mercury were lost during gold mining in Brazil (in the form of both emissions to the atmosphere and release in rivers and soils) [21].

While the repression of illegal activities can imply the imposition of fines related to damages, there are still important methodological bottlenecks to establish responsibilities, and no standardized approach to evaluate damages. Methodological bottlenecks depend on several factors. Firstly, it is necessary to differentiate between natural mercury (i.e., particles mineralized present in soil and sediment) and additional mercury from human activities (i.e., mercury that is intentionally released in the environment) [22,23,24,25,26,27]. Secondly, there are other potential sources of mercury, such as deforestation from agriculture and cattle ranching [28,29,30,31,32,33,34]. Although some studies argue that these activities can have a greater aggregate impact on mercury release into the environment than small-scale gold mining [35,36,37,38,39,40,41,42,43], global statistics indicate that 30% of mercury in the environment results from anthropogenic activities, of which small-scale gold mining accounts for 37% of all releases and is a major source of contamination [44]. The third factor is related to the difficulty in attributing responsibility to specific mines. Although there is evidence of increased mercury concentration in the Amazonian population, this increase is generated by the combined effect of several illegal gold mines. Moreover, besides the mines existing today, there are mines that ceased to exist decades ago, leaving a cumulative impact [45,46]. Therefore, the accountability of specific gold mines has become a great challenge given the complexity of the mercury cycle.

The mercurial form used in the gold ore extraction process is metallic mercury, also known as elemental mercury (Hg^0^). The fraction of metallic mercury that is not recovered during the extraction process contaminates the atmosphere and rivers in the Amazon region. Once released into aquatic systems, a part of metallic mercury is oxidized and can be methylated by the action of microorganisms or abiotic factors. This process gives rise to the most dangerous mercury species, methylmercury (MeHg). Methylmercury is biomagnified along the aquatic food chain, contaminating fish and other organisms used for food such as turtles, crabs, shrimp and alligators. Furthermore, much of the toxicity of methylmercury is due to its high neurotoxic potential and its ability to overcome the blood-brain and placentary barriers.

A vast body of literature has analyzed the increase in contamination levels in the Amazon population [47,48,49,50,51,52,53,54,55,56,57,58]. Vasconcellos et al. [49] found an average methylmercury hair level of 7.0 μg/g in the Munduruku indigenous community in Tapajós and Vega et al. [58] observed that the Yanomami indigenous community, also in Brazil, had hair methylmercury levels higher than 6.0 μg/g, which is far above the maximum recommended level of 1.0 μg/g by the United States Environmental Protection Agency (U.S.EPA) [59] and 2.3 μg/g recommended by The Food and Agriculture Organization of the United Nations (FAO/WHO) [60]. Despite evidence of the contamination of the population, to the best of our knowledge, no study in the world has attributed the relative share of responsibility of artisanal and small-scale gold mining (ASGM) extraction to the health impacts of the population.

On the other hand, the available literature has extensively documented the impact of mercury on health outcomes. For example, the cohort studies conducted in the Faroe Islands and New Zealand indicate that even in low doses, the consumption of mercury-contaminated fish during pregnancy can cause important cognitive alterations in children [61,62]. In this sense, mercury’s potential neurotoxic effects in children and adults of the Amazon have been analyzed in some studies [63,64,65,66,67,68]. The most common effects in children are cognitive problems, neurodevelopmental impairment, and psychomotor disorders. Depending on the mercury exposure level in the prenatal period, the child may be born with mild mental retardation. Axelrad et al. [69] showed that for each additional 1.0 µg/g of methylmercury in maternal hair a reduction of 0.18 IQ points is expected in the child. According to Vasconcellos et al. [10], the methylmercury hair concentrations detected in women of reproductive age in the Amazon region are high enough to cause the emergence of cases of mild mental retardation. In adults, decreased visual field, neurobehavioral, and motor coordination disorders are most frequently reported [68]. Meanwhile, literature on the impact of mercury on increases in cardiovascular disease is still not unanimous [70,71], but there is evidence of this relationship in non-Amazonian countries [8,9,47,48]. Salonen et al. [8] showed an increased risk of myocardial infarction of 69% in men over 40 years when hair mercury levels were above or equal to 2.0 µg/g, compared to men with levels below to 2.0 µg/g.

Several studies have addressed the relationship between extracted gold kilograms and effects on human health [72,73,74,75,76]. For evaluating public policies associated with mercury impacts on human health, we used a combination of non-monetary indicator–disability adjusted life years (DALY) [77,78,79] and monetary indicator–value of statistical life (VSL) [80], based on willingness to pay for risk reduction [81,82]. Many studies use this relationship between VSL and DALY. Neumann et al. [83] review studies that depart from the analysis in DALY to assess the impact through a monetary indicator. Fan et al. [84] estimate COVID-19’s impact from DALY and Statistical Value of Life. Grandjean and Bellanger [85] calculate disease burden associated with environmental chemical exposures, including methylmercury worth US$15 billion for Europe and the United States.

Many authors have studied methylmercury ingestion by humans via contaminated fish consumption, analyzed the impact of this contaminant on human health, and established relationships between average mercury intake and negative effects on human health [8,10,47,48]. However, these studies do not distinguish the potential origin of mercury (i.e., whether it is natural or additional mercury). For this reason, we sought to develop a methodology that would allow us to analyze mercury dispersal by gold mines, making it possible to link the human health impact per gold mine from the input unit, such as impacted hectares or gram of gold extracted.

The main literature gaps addressed here are: (i) the potential area of mercury dispersal in the water, as well as the estimation of the maximum affected population, given the average level of contamination and fish consumption; (ii) the definition of dose-response functions for different health outcomes and population groups; and (iii) the valuation of health impacts of mercury in monetary terms. Therefore, we seek to link a chain of events, concepts, and value estimates from the literature through a series of hypotheses, which allow us to relate the use of mercury in small-scale gold mines to health outcomes and their negative economic values.

Considering all of the gaps mentioned, the main objective of this study was to develop a precise methodology to estimate the health damage caused exclusively by the mercury used in ASGM (that is, avoiding counting the natural mercury present in Amazonian soils). For estimation calculations, the amount of gold that is extracted in a given location and the amount of mercury used and discarded during the process were considered. The study provides value estimates that can support social impact assessment, define fines for illegal gold miners, and provide parameters for the evaluation of public policies related to inspection and prevention of this activity. The methodology construction contributes to the incorporation of impacts on human health by decision makers and expands the discussion on the effects of illegal mining activities in a strategic area such as the Amazon for economic development. This study is the first to estimate the average impacts that a gold mine can generate on human health due to the use of mercury and its ingestion from fish consumption.

## 2. Materials and Methods

This section, based on an extensive literature review, establishes the hypotheses to make the connections between average mercury use, its dispersion in the environment, potential absorption by humans, and its effects on human health. The study area is the Brazilian Amazon, which concentrates 93% of all small-scale mining in Brazil [86]. Gold mining is more concentrated in the states of Pará and Mato Grosso, in addition to significant impacts in the state of Roraima, and more specifically in Yanomami Indigenous Land.

We establish links between several factors, using average values from the literature, in a chain which can be divided into two major goals: how mercury used in gold mining is dispersed in water until it reaches humans, and the quantification of the impact on human health from mercury ingestion.

**Figure 1:**
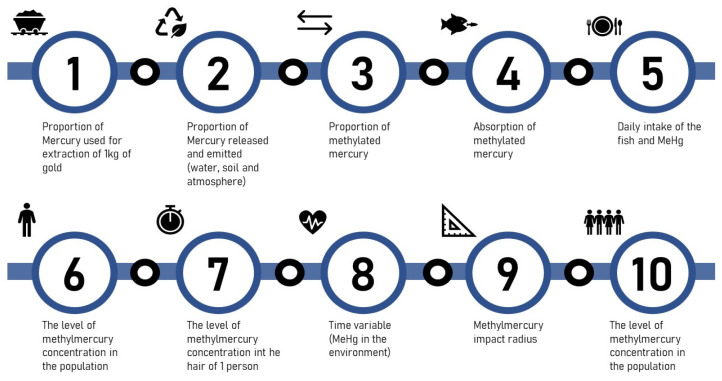
Logical line for relating the existence of gold mining to human health outcomes.

To complete the logical line presented in Figure 1 below, an extensive bibliographic review was carried out on various topics such as biophysics, biochemistry, epidemiology, and public health.

The study begins with the presentation of the relationship between the use of mercury from gold mines and the effects of increasing methylmercury concentration in the hair of the affected population. Then, the study assesses which adverse effects on human health are considered when there is an increase in the level of methylmercury in the hair.

### 2.1. Dispersion of Mercury Used by Gold Mining and the Extent of Exposure and the Health Risk the Affected Population

We aim to relate the use of mercury by gold mining with the increased mercury concentration in the hair of the affected population. We divided into two main specific objectives. The first is to explain how the mercury used in gold mining is dispersed in water until it reaches humans. The second is to describe how mercury from gold mining and human mercury intake affects the population.

#### 2.1.1. Variables That Define How the Mercury Used in Gold Mining Is Dispersed in the Water until It Reaches Humans

The following variables have the objective of explaining which variables are important in defining how the mercury used in gold mining is dispersed in the water until it reaches humans. For this, the next three described variables are the proportion of mercury used for each kilogram of mined gold, the proportion of mercury released in water and soil and emitted into the atmosphere, and the proportion of mercury that transforms into methylmercury (methylation). The last section presents a summary of these variables.

##### Proportion of Mercury Used for Each Kilogram of Mined Gold

Mercury is used in the amalgamation process, which, when combined with gold, forms a metallic alloy called amalgam. The literature shows that the proportion of mercury used in the extraction process can vary in both Brazil and worldwide due to different factors (such as different yields for gold extraction). This proportion can vary considerably from 1.3–8.0 g of mercury for each g of gold extracted [17,72,74,87,88,89]. An average ratio (Hg:Au) of 2.6:1 demonstrated by Castilho and Domingos [17] is assumed here, since it was obtained as an average from different gold mines in Brazil.

##### Proportion of Mercury Released in Water and Soil and Emitted into the Atmosphere

The mercury used in gold mining is dispersed in both soils and rivers, as well as in the atmosphere. This study analyzes only the release of mercury in aquatic environments, since the objective is to understand the effects of methylmercury on human health from ingesting contaminated fish. Therefore, we sought to review the literature that indicates the proportion of mercury released in soil and water. This proportion can vary from 12–35%, with the lowest proportion (12%) being a conservative scenario [17,18,19,20,88]. It is important to mention that such studies already consider environmental controls such as filtering in the amalgamation process (which recovers 50% of the mercury).

##### Proportion of Mercury That Transforms into Methylmercury (Methylation)

When mercury is dumped into an aquatic environment, part of it is transformed into an organic mercury compound called methylmercury, which is about 30 times more toxic than inorganic mercury [90] and is ingested via the consumption of contaminated fish. Once ingested by humans, methylmercury is rapidly absorbed by the gastrointestinal tract and widely distributed throughout the body, including reaching the central nervous system, which can cause IQ loss in children and cardiovascular diseases [8,9,10,47,48,72,73,74,75,76].

Given the knowledge of the amount of mercury released into water, another process widely discussed in the literature is the proportion of mercury that transforms into methylmercury (MeHg).

The literature shows that the methylation process occurs in different contexts in the Amazon, with conditions such as low pH and high levels of dissolved organic carbon favoring mercury oxidation and methylation [90]. Several studies have shown that the methylation rate can vary between 3% and 22% [91,92,93,94,95,96,97]. Conservatively, the choice for the lowest methylation rate indicates that 3% of mercury released into the water will change to methylmercury over time.

##### Variable’s Interaction to Explain How Mercury Used in Gold Mining Is Dispersed in the Water until it Reaches Humans

The formula below demonstrates the results found from Section 2.1.1:

(1) X= A∗B∗C∗D

where:

X = mercury used by mining, is released into aquatic environments and undergoes methylation

A = gold amount extracted by mining (kilos)

B = proportion of mercury used for extraction of each kilogram of gold (%)

C = proportion of mercury released in the water (%)

D = methylation rate (%)

#### 2.1.2. Variables That Describe How Mercury from Gold Mining and Human Mercury Intake Define the Affected Population

After estimating the amount of mercury that is dispersed in the environment and potentially consumed by humans, we estimated how this amount will be consumed: (i) by individuals who eat contaminated fish daily; and (ii) by the number of individuals that may absorb this amount of mercury across their lives. Therefore, we need to understand how many people may be exposed (up to an average contamination level) by a consuming a given additional amount of mercury throughout their lifetime.

The average long-term contamination level of one individual is estimated based on the daily contaminated fish consumption, which gives us the total amount of mercury that will lead one individual to the negative health outcomes considered. Based on this individual total consumption, we can estimate how many individuals could be impacted at the same contamination level within a given impact area.

##### Methylmercury Absorption by Fish

Methylmercury is a chemical substance that is absorbed by the trophic chain in aquatic environments [98]. Through biomagnification, substances or elements in living organisms travels through the food webs and accumulates at the highest trophic level, differing between predatory and non-predatory species [99,100,101].

As an organic form of Hg, MeHg has extremely neurotoxic effects and is readily accumulated in biota due to its lipophilic and protein-binding properties [102,103]. A series of studies have shown the average levels of contamination of aquatic species in the Brazilian Amazon [49,52,53,54,55,56,57,58,104,105,106,107,108,109]. For example, contamination can reach 0.13 to 2.85 μg Hg/g for certain fish species [104] and Dórea et al. [55] detected mean mercury levels of 0.578 g Hg/g piscivorous fish and 0.052 g Hg/g non-piscivorous fish in the upper Tapajós basin.

To use recommended values at a global level, this study chose the Codex Alimentarius [110], jointly developed by the Food and Agriculture Organization of the United Nations (FAO) and the World Health Organization (WHO). According to Codex [110], the maximum permitted mercury levels are 1.0 μg/g and 0.5 μg/g for predatory and non-predatory fish trade, respectively.

##### Average Daily Intake of Fish and Methylmercury for Different Profiles in Brazil

The Amazon region is historically known for eating fish, whether in riverside and indigenous populations or large urban centers. Riverside dwellers, for example, eat more fish, with an average consumption of 189 to 243 g of fish per day [18,49,50,109,111,112,113]. Meanwhile, the indigenous population have an average daily fish consumption of 100 g per day [49] from fishing. Finally, the urban population, such as in Belém city (Pará state), have a more diversified diet with other proteins and, therefore, consume an average of 57 g per day [114].

To calculate the average daily mercury intake per person, we described the average weight of an individual in each population profile as 70 kg for urban people [115], 65 kg for riverside dwellers [49], and 53 kg for indigenous people [50].

Chronic ingestion of mercury-contaminated fish by the Amazon population increases health risks.

The formula below represents how average daily intake is calculated:

(2) $$I=P*\left[\frac{\mathrm{Cm}*\mathrm{Cont})}{W}\right]$$

where:

I = average mercury daily intake (μg/kg bw (Bw = body weight)/day)

P = proportion of urban, indigenous, and riverside populations in the municipality affected by gold mining. Therefore, a balance between these factors is necessary.

Cm = average fish consumption per day (g/day) for population type

Cont = average contamination in fish (µg/g fish) (depends on fish absorption of methylmercury presented in Section 2.1.2)

W = individual weight (kg) for type of population

The average daily intake is related to contextual factors of the location that is being affected by gold mining, such as the proportion of indigenous, riverside, and urban populations.

##### Variable Time for Methylmercury Ingestion by Fish and Humans

Several studies have demonstrated the instability and complexity of methylation and demethylation [91,92,93,94,95,96,97]. Bisinoti and Jardim [116] demonstrated that all mercury-containing river and lake sediments are dangerous since confined mercury can remain active as a substrate for methylation for approximately 100 years. Thus, the conservative hypothesis is that mercury is bioavailable in the environment for 50 years and can cause harm to human health for this duration.

Based on this hypothesis, it is possible to quantify the mercury consumption for an average individual over 50 years using the following formula:

(3) Z=Y∗T

where:

Z = average mercury intake per person over 50 years (g/50 years per person)

T = time (50 years = 18,250 days)

Y = average mercury daily intake per person (g/day per person) (conversion based on average individual weight (kg) and average mercury daily intake (μg/kg bw/day)

Therefore, it is assumed that the daily mercury intake by each social group in the region (riverside, indigenous, and urban population) will chronically occur over 50 years. In other words, individuals with an average daily intake of 0.76 g/kg/day will have a total intake of 0.9 g over 50 years, resulting in an average increase in mercury contamination of 5.0 µg/g µg/g (hair). Such information is essential to understand how methylmercury released by gold mining will be distributed among fish consumers in the region.

Knowledge regarding mercury release by gold mining and subsequent impacts on fish and daily mercury intake by humans, when associated with the time variable, requires a hypotheses on the proportion of methylmercury that will be absorbed during this period. We hypothesize that all bioavailable methylmercury from gold mining will be consumed by humans, that is, will be consumed between 0.22% and 4.5% of the total mercury used by ASGM. This hypothesis does not imply that all methylmercury will be instantly absorbed by humans, but rather chronically absorbed over 50 years by humans at the top of the trophic chain. This type of hypothesis is needed due to the literature gap on tracing mercury molecules from ASGM until human absorption.

##### Changes in the Methylmercury Hair Concentration Level

Methylercury will accumulate in the hairs of people who consume of fish contaminated by mercury. This clear relationship between contaminated fish consumption and methylmercury accumulation in hair was demonstrated by the Poulin and Gibbs [11]. According to the World Health Organization [117], an average daily intake of 0.1 μg/kg of methylmercury leads to a concentration of 1.0 µg/g of methylmercury in hair. Thus, it is possible to estimate how gold mining influences methylmercury concentration in hair.

(4) $$F=\frac{I}{0.1}$$

where:

F = hair methylmercury concentration (in ug/g)

I = average mercury daily intake (μg/kg bw/day)

##### Population Impacted by Increased Methylmercury Concentration in Hair

Knowledge about the amount of mercury released by gold mining, methylated in the aquatic environment (Section 2.1.1), and distributed over time (50 years) with the average intake per person (grams of mercury in 50 years) (Section 2.1.2), contributes to defining the population affected by mercury used in gold mining as the formula can be presented as follows:

(5) $$\mathrm{Pop}=\frac{X}{Z}$$

where:

Pop = population affected by mercury contamination from gold mining

Z = average mercury intake per person

X = estimated amount of methylmercury from mining that reaches the top of the trophic chain.

Socioeconomic characterization of the affected population is considered, where groups with higher fish consumption are expected to have higher contamination values. The average daily intake of mercury through fish consumption is differentiated by the riverside population, which has a higher fish consumption, and urban population, having a lower fish consumption and a lower chance of being exposed.

Additionally, we considered the population density as a limiting factor of the total number of people that may be exposed within a given radius. Mercury distribution in river and tributaries is influenced by the distance from gold mines [118]. Therefore, we stipulate a limit for mercury impact that depends on the population size in the neighboring mining area. The amount of mercury close to the source of contamination is high and decreases as the analysis distance increases, indicating low concentrations far from the analysis point [119,120]. Studies have also shown that, when assessing the amount of mercury in cities close to gold mining areas, the mercury concentration in the hair of the population living near the mines was greater than in people living far from the gold mines [121,122]. However, river confluence events, where one river flows into another (whose mercury concentration is higher), may indicate a pattern of increasing mercury concentration after a certain distance [123,124].

Several studies present the average distance that metallic mercury can travel in rivers, ranging from 4 to 100 km [36,124,125,126,127,128,129,130], as a function of river characteristics (flood events, rain, and increased water flow). In the Amazonian context, studies such as Roulet et al. [36] have shown that the significant impact radius is approximately 50 km downstream. However, it should be noted that organic mercury (methylmercury) can travel longer distances as it is absorbed by fish, which can migrate up to 2000 km, such as Brachyplatystoma (*Piratinga*) [131,132,133,134,135,136]). Therefore, we conservatively assumed that mercury will be dispersed to a radius of 100 km (that is, we did not consider the long distances traveled by some species of fish since there is heterogeneity of migration depending on the species and it would be complex to standardize for the Amazon region).

The effects of this release are limited to the number of people within a 100 km radius. A highly contaminated region with a low population density means that few humans will be affected, although it continues to have a significant impact on the region’s fauna. Likewise, urban areas close to the center of contamination have a high population density, potentially causing damage to human health of more individuals.

##### Variable’s Interaction to Explain How Mercury Used in Gold Mining Is Dispersed in the Water until It Reaches Humans

The formula below demonstrates these results found from Section 2.1.2:

(6) $$F\;=\left[\frac{\left(\mathrm{Cm}*\mathrm{Cont}\right)}{W}\right]*\frac{P}{0.1}$$

where:

F = hair methylmercury concentration (in ug/g)

I = average mercury daily intake (μg/kg bw/day)

P = proportion of urban, indigenous, and riverside populations in the municipality affected by gold mining. Therefore, a balance between these factors is necessary.

Cm = average fish consumption per day (g/day) for population type

Cont = average contamination in fish (µg/g fish) (depends on fish absorption of methylmercury presented in Section 2.1.2)

W = individual weight (kg) for type of population

#### 2.1.3. Mercury Dispersion Summary Formula

All of the logical links described above can be summarized as follows. It is possible to relate mercury use by gold mining and its respective loss in the environment until it reaches the human body and affects the population:

(7) $$\mathrm{Pop}=\frac{X\;\left(A,\;B,\;C,\;D\right)}{Z\;\left(\mathrm{Cm},\;\mathrm{Cont},\;P,\;W,\;I,\;T\right)}$$

where:

Pop = population affected by mercury contamination from gold mining

X = mercury used by mining, is released into aquatic environments and undergoes methylation (depends on the proportion of mercury used for extraction of each kg of gold, and the proportion of mercury released in the water; the methylation rate

Z = average mercury intake per person in 50 years (g/50 years per person) (depends on the absorption of methylated mercury (100%).

A = gold amount extracted by mining (kilos)

B = proportion of mercury used for extraction of each kilogram of gold (%)

C = proportion of mercury released in the water (%)

D = methylation rate (%)

I = average mercury daily intake (μg/kg bw/day)

P = proportion of urban, indigenous, and riverside populations in the municipality affected by gold mining. Therefore, a balance between these factors is necessary.

Cm = average fish consumption per day (g/day) for population type

Cont = average contamination in fish (µg/g fish) (depends on fish absorption of methylmercury presented in Section 2.1.2)

W = individual weight (kg) for type of population

T = time variable (MeHg in the environment) = 50 years (multiplied by mercury intake per person).

### 2.2. Quantifying Impacts on Human Health from Mercury Ingestion

The health economics literature quantifies the impact on health using the disability adjusted life years (DALY) index to compare the impact of different health problems. The DALY index weighs health measures of mortality and morbidity in one equivalent measurement unit: time (years), considering the severity, magnitude, and duration of the problem [137]. Different knowledge areas are considered to quantify the gold mining mercury impact in terms of increases in the probability of developing: (i) mild mental retardation in children, (ii) myocardial infarction, and (iii) hypertension.

To calculate the DALY, it is necessary to know the following variables: discount rate, age, weight, disability weight, disease duration, and incidence rate which can be seen in the next sections.

#### 2.2.1. Mild Mental Retardation Impact Caused by IQ Loss in Children

The next section relates mercury release in gold mining to IQ loss in children and mild mental retardation due to maternal ingestion of contaminated fish, a health outcome that leads to loss of productivity and income from the birth of the infected child to death [11]. Axelrad et al. [69] demonstrated a linear relationship between loss of points on the IQ scale and increases in mercury concentration in maternal hair, in which 1.0 μg/g of mercury (MeHg) in the mother’s hair corresponds to a loss of 0.18 IQ points in the child. Considering that IQ values in the general population have a normal distribution (Gaussian curve) and 95% of individuals have IQ values between 70 and 130, the IQ loss caused by mercury exposure during the prenatal period may cause mild mental retardation in individuals who would be born with IQ values close to 70.

Given the total affected population, as described in Section 2.1.3, we use the number of live births of 19 live births per thousand inhabitants in the North of Brazil. That is, out of a population of 1000 affected people within a radius of 100 km, around 19 babies will be born alive. It is possible to estimate the number of live births impacted by mercury release in the mine.

To calculate the DALY related to IQ loss in children, we highlighted the variables based on a literature review on the subject. One of these parameters is a discount rate which can be defined with the objective of assigning less importance relative to years lost in the future than to years of life lost in the present, given that a human being, in general, has short-term rather than long-term preferences [138,139]. 

We chose to use the 3% discount rate as it is applied in health economics studies [140], in environmental projects [59], in the calculation of the social cost of carbon [141] and in for social projects in Latin American countries [142]. Another variable is the age weight that is the age weight that corresponds to society’s preferences, since less value is given to healthy years of life lost during childhood and old age, due to the low productivity common to these stages of life. The weight of age varies in a range from zero (without weight) to one (100% of weight), being relevant as a weighting factor so that greater weights are not attributed to cases of death in young individuals. The third parameter is the incidence rate (number of cases per thousand people) which is calculated by the Mercury Spreadsheet [11] from the knowledge of the mean concentration of mercury in the hair and the standard deviation associated with the knowledge of the number of affected people). The fourth variable for calculating the DALY is the disability weight which is the result of some studies that create scenarios for individuals to declare their preferences and, therefore, the different outcomes are compared by patients or specialists, creating a ranking [143]. The disability weight can range from 0 to 1, where 0 is a healthy situation and 1 corresponds to death. In the specific case of mild mental retardation in children due to mercury ingestion, according to the WHO [143], the weight was 0.361. The fifth parameter is the year onset of disability and duration that are fundamental in weighing the impacts, since years lived with disability or premature death are counted. In the specific case of IQ loss, the outcome starts in the first year of the child’s life and remains throughout life. As in the North region of Brazil, there is a life expectancy of 72 years, thus meaning a disease duration of 72 years.

The monetary measurement of DALY has been widely discussed in several studies, such as Kenkel [82] and Hammit and Robinson [144], who proposed that 1 DALY corresponds to the annualized value of statistical life. This means that the monetary measurement of DALY can reach values above 200,000 USD per DALY [145]. We use the recommendation of the World Health Organization [146], which suggests that one year of healthy life lost (DALY unit) corresponds to 3 GDP per capita, that is, 20,600 USD in Brazil in 2020 [147]. Thus, the mild mental retardation in children due to the extraction of 10 kg of gold corresponds to 10,000 USD in Brazil if all of the average values described throughout the paper are observed.

#### 2.2.2. Cardiovascular Diseases

The association between contaminated fish consumption and cardiovascular diseases considers that mercury in fish muscle, when absorbed by the human gastrointestinal tract, interferes with lipid peroxidation and can cause atherosclerosis. This condition can lead to increased blood pressure [47] and acute myocardial infarction [9]. On the other hand, some studies have not found a relationship between mercury and cardiovascular disease, although they suggest the need for studies on such a relationship [148,149,150,151,152].

This section presents the parameters used to describe the relationship between mercury concentration in hair due to the use of mercury in gold mining and two cardiovascular diseases: acute myocardial infarction and arterial hypertension.

##### Acute Myocardial Infarction Attributable to Mercury Exposure

For acute myocardial infarction, Salonen et al. [8] found that an individual with a hair mercury concentration of ≥2.0 μg/g has a 69% higher risk of acute myocardial infarction than individuals with a concentration of less than 2.0 μg/g. This relative risk presented by Salonen et al. [8] was adjusted for confounding factors, such as alcohol consumption, smoking, and lifestyle factors, and refers to the probability of incidence of acute myocardial infarction, fatal or non-fatal, in Finnish men over 40 years.

1. Calculating Acute Myocardial Infarction Burden Disease Attributable to Mercury Ingestion from Gold Mining

The disease burden methodology in the gold mining context in the Amazon was adapted from a study by Salonen et al. [8]. We first calculated the attributable fraction based on the relative risk estimated by the study of Salonen et al. [8] in Finland. Studies such as Rockhill et al. [153], Fewtrell et al. [149], and Porta [154] presented formulas to estimate the attributable fraction from the relative risk, calculated at 1.69, in Salonen et al. [8] for an exposure to mercury above 2 µg/g. Based on this, we estimated that the risk of myocardial infarction occurrence [9] is 0.4, that is, 40% of myocardial infarction cases can be attributed exclusively to mercury exposure ≥2.0 µg/g. This paper assumes as a hypothesis, based on evidence from field measurements [10,49,50], that, due to the high mercury intake, the entire affected population will be at risk of an average mercury concentration above 2.0 µg/g.

To estimate the “Number of Infarction Cases Attributable to Mercury Exposure (≥2.0 µg/g Hg)” it is necessary to multiply the total number of infarcts in the sample and the attributable fraction.

(8) $$\mathrm{Ip}=\frac{\mathrm{Afi}}{\mathrm{Ti}}$$

where:

Ip = people infarcted due to mercury levels > 2.0 µg/g

Afi = attributable fraction (infarction

Ti = total number of infraction cases

The same gender and age cut out made by Salonen et al. [8] was used: men over 40 years, which represents 12% of the population of the Brazilian Amazon [155]. Therefore, if data from Datasus [156] are observed, in 2015–2020, approximately 0.16% of this population would be at risk of hospitalization due to infarction in the North region.

As the present study uses the study by Salonen et al. [8] as a basis, we opted for the conservative premise that the year onset of disability is equal to the youngest age of the sample in Salonen et al. [8] (that is, 40 years old). It should be noted that data from Datasus [156] are probably underestimated since the Brazilian health system cannot compute information from isolated areas in the northern region of Brazil.

To adapt to the Amazon context the regional life expectancy was set as 67 years for men. As we consider that the average age of the infarction is 40 years, this means that individuals will live with a disability from 40 years of age to 67 years of age (that is, they will have lived with such disability for 27 years).

**Figure 2:**
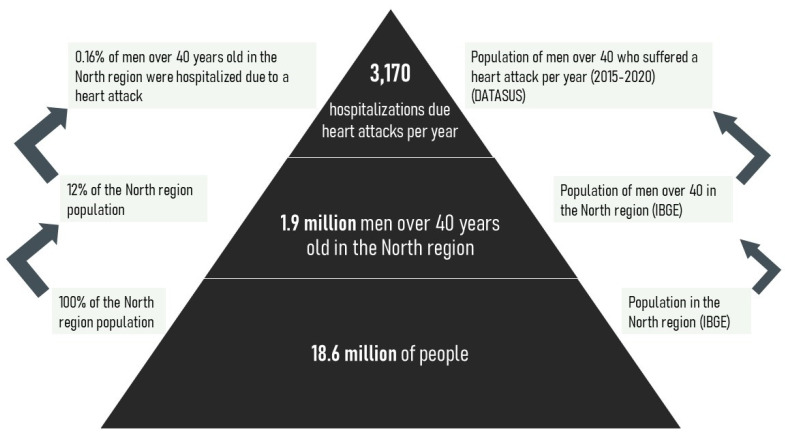
Outcome of myocardial infarction associated with mercury ingestion.

Figure 2 demonstrates the logical chain built above:

Values for the probability of risk accumulated over the years are based on Zaletel-Kragelj and Bozikov [157], who estimated the cumulative risk of mercury-associated myocardial infarction at 1.61%.

The estimation of the number of infarction cases associated with mercury over the years for the male population over 40 years old and the accumulated infarction risk associated with mercury over time is multiplied.

(9) Hm=Cri∗Mp

where:

Cri = cumulative risk of mercury-associated myocardial infarction = 1.61%

Hm = male population over 40 in the region who will be hospitalized in 27 years for mercury ingestion

Mp = male population over 40 years in region

2. Variables for Calculating the DALY and Monetary Impact of Mercury-Associated Acute Myocardial Infarction from Gold Mines

The DALY for mercury-associated acute myocardial infarction was based on the following parameters: 3% discount rate, 100% weight for age, disability weight for acute myocardial infarction of 0.439 [143], disability onset at age 40, disability duration of 27 years (assuming life expectancy of 67 years), and an incidence rate of 1.9 cases of infarction for every 1000 people.

The resulting value is given in years lived with disability for the extraction of gold per kg. For example, 10 kg of gold can generate, on average, the impact of 8.5 years lived with disability or 174,000 USD at 20,600 USD for each DALY [147].

##### Arterial Hypertension Disease Attributable to Mercury Exposure

High blood pressure has long been recognized as a major risk factor for cardiovascular diseases. A recent analysis suggests that the burden of high blood pressure has increased over the past three decades [158,159]. In addition to traditional risk factors for hypertension, such as high salt intake and overweight/obesity, environmental exposure to heavy metals can also play an important role [160,161,162]. Although the mechanisms by which mercury induces hypertension are not fully understood, plausible explanations include oxidative stress and inflammation, which promote endothelial and renal dysfunction and binding of selenium-related enzymes. Hu et al. [47] included a systematic review, building a meta-analysis both with general studies and with the occupational population exposed.

1. Methodology for Calculating the Hypertension Burden Disease Attributable to Mercury Ingestion from Gold Mining

The hypertension disease burden methodology in the context of gold mining in the Amazon fundamentally involves adaptation to the study by Hu et al. [47], with the definition of all applied premises being relevant. The first adaptation to the study by Hu et al. [47] consists of the estimate of the attributable fraction from the odds ratio (OR) of 1.35, given by the meta-analysis for mercury exposure. Since the OR is analogous to the relative risk, it is assumed that they are similar, as shown in studies such as Bonita et al. [163].

Although Hu et al. [47] presented studies for the Amazon context, such as Fillion et al. [9], with an OR of 3.8, indicating a high concentration of mercury in the Brazilian Amazon population), we adopted, conservatively, the OR of the meta-analysis, that is, 1.35, since this is a comprehensive study review on the relationship between hypertension and mercury intake. Therefore, it is possible to quantify the attributable fraction using the following equation:

(10) $$\mathrm{FAP}=\frac{\left(\mathrm{OR}-1\right)}{\mathrm{OR}}$$

Based on this understanding, we estimated the risk of arterial hypertension occurrence [47] to be 0.26, that is, 26% of cases of arterial hypertension would be due exclusively to mercury exposure ≥ 2.0 µg/g.

To estimate the “Number of Hypertension Cases Attributable to Mercury Exposure (≥2.0 µg/g Hg)” we multiplied the total number of hypertension cases in the sample by the attributable fraction.

(11) Hp=Afh∗Th

where:

Hp = number of hypertension cases attributable to mercury exposure (≥2.0 µg/g Hg)

Afh = attributable fraction (hypertension)

Th = total number of hypertension cases

**Figure 3:**
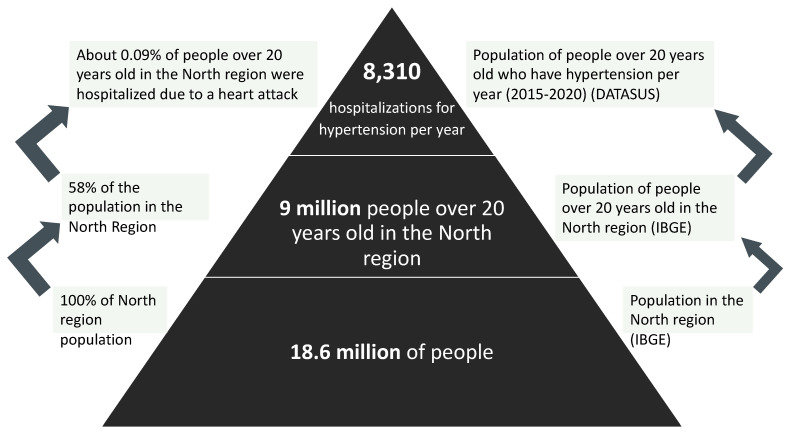
Hypertension scenario in Northern Brazil.

Unlike the myocardial infarction outcome, the literature does not indicate a greater or lesser hypertension risk depending on gender (male or female); that is, the population over 20 years should only be evaluated as that is the year in which hypertension begins to be observed [47]. Figure 3 summarizes the logical lines built above.

To attribute the fraction of this outcome to fish intake, it is necessary to use the attributable fraction calculated as 26% of the risk associated with mercury. Using the methodology of Zaletel-Kragelj and Bozikov [157], the cumulative hypertension risk associated with mercury was estimated to be 1.21%.

Based on the knowledge of the temporality of the outcome, it is feasible to estimate the number of cases of hypertension associated with mercury over the years. For this, the population over 20 years in the region that will be hospitalized over 52 years of exposure and the accumulated risk of hypertension associated with mercury over time is multiplied.

(12) Hp=Crh∗Pp

where:

Crh = cumulative risk of mercury-associated hypertension

Hp = population over 20 years in the region who will be hospitalized in 52 years for mercury ingestion

Pp = population over 20 years in region

2. Variables for Calculating DALY and Monetary Impact of High Blood Pressure Associated with Mercury from Gold Mining

To calculate the DALY related to arterial hypertension, we considered the following parameters: discount rate of 3%; 100% weight for age; disability weight of 0.246 [143]; year onset of disability at the age of 20 years, with a duration of 52 years to meet the 72 years of life expectancy in the northern region of Brazil.

## 3. Results

### 3.1. Results Presented from Methodology

The methodology presented above consists of the first estimate of the relationship between the use of mercury by gold mining and the negative effects on human health. Defining the amount of mercury used per kilogram of gold mined (Section 2.1.1), the proportion of mercury loss to the environment (Section 2.1.1), and the methylation rate (Section 2.1.1) means that, on average, between 0.22% and 4.5% of the total mercury used by gold mining turns into methylmercury, entering the trophic chain and therefore affecting human health. Such an amount may seem small, but the effects on human health are varied and extremely harmful. The result is the first estimation that quantifies the release of mercury into the water, which contributes to combat arguments such as that deforestation, is the main cause of the release of natural mercury into the environment.

Given the knowledge of the amount of mercury released by gold mining, the present study demonstrated that the population context is essential to define the impacts on human health. Therefore, it is assumed that the daily mercury intake depends on social group affected in the region (e.g., riverside, indigenous, and urban population) As an example of a population with indigenous and riverine population, an average daily intake of 1.2 μg/kg bw/day corresponds to an average concentration in hair of 12 µg/g, which corroborates other studies. Bastos et al. [18], identified an average mercury concentration of 9.81 µg/g in 45 riverine communities. Vasconcellos et al. [49] detected mercury levels above 6.0 µg/g in hair samples in Munduruku indigenous communities in the Pará state. However, in urban populations, a daily mercury intake of 0.4 μg/kg bw/day corresponds to an average concentration of mercury in hair of 4.0 µg/g; greater than the 1.0 μg/g recommended level by the North American Environmental Protection Agency [59] and 2.3 μg/g by the United Nations Food and Agriculture Organization [60].

Likewise, the study demonstrated that the context of the affected population is not restricted to these population characteristics, it is also important to consider population density and the distance of the population to the gold mining. This is explained due to the fact that areas with low population density and far from gold mining will have a limited effect on the health of the population, while regions with high population density and close to a radius of less than 100 km has a higher probability of consumption the methylmercury released by the ASGM.

The Table 1 below seeks to summarize the ranges between the variables used in the model:

By defining the characteristics that influence the level of contamination and the number of people affected, the study made use of existing studies that addressed the relationship between exposure to mercury and impacts on human health [9,10,47]. In the outcome of mild mental retardation in children, there is already wide acceptance about the relationship between level of contamination and lost IQ points [69]. Therefore, the study applied the methodology already developed by Poulin and Gibb [11] that uses the DALY indicator to quantify the loss of well-being.

Meanwhile, for cardiovascular outcomes (arterial hypertension [47] and myocardial infarction [9]), adaptations were necessary since there is no direct relationship between the level of contamination and the impact on human health. Therefore, deepening the theme of epidemiology was necessary, being one of the contributions of the article.

As a result, in a population of 100,000 impacted people, around 193 men over 40 years of age will have a myocardial infarction associated with mercury ingestion. The incidence rate of this outcome is given by the number of cases in every 1000 affected people. That is, 1.9 hospitalization cases for infarction associated with mercury in every 1000 affected people. Similarly, the results in an affected population of 100,000 people will average about 700 people with high blood pressure in the population over 20 years of age.

### 3.2. Human Health Impact in the Indigenous Territory Due to Illegal Gold Mining

The methodology was applied to evaluate the negative impacts of illegal miners occupying the Yanomami Indigenous Land (YIL), an area located mainly in the Brazilian Amazon in the states of Roraima and Amazonas. YIL is the largest indigenous land in Brazil, with an area of 96,000 km^2^ and a total population of 26,780 indigenous people. Although the Brazilian constitution prohibits economic activity on indigenous lands, the main potential threat faced by YIL is invasion by illegal miners. It is estimated that more than 25,000 miners live and work illegally in the territory.

The variables used to present the results are described in the following Table 2.

Despite the variability of each parameter shown in Table 1, we considered conservative parameters in the literature in Table 2, showing that the risk to human health can potentially be greater than what is being presented with this estimate. A more pessimistic scenario, following the precautionary principle, with higher parameters, demonstrates a potential for greater harm to the population’s health.

In 2020, 5 km^2^ were degraded by illegal ASGM [165], which, considering average productivity of 1.7 kg of gold per hectare in Brazilian Amazon [166], would use around 2.2 tons of mercury for 863 kg of gold production. As a result, we estimated that approximately 32 kg of mercury was released into local rivers, which could affect 44,000 people. Using our methodology, we estimated that 307 people would develop hypertension problems, 85 acute myocardial infarction, and 4 mild mental retardation. The economic value of these human health damages would total 69 million USD, divided into: (a) 1 million USD due to IQ loss; (b) 15 million USD due to acute myocardial infarction; and (c) 52 million USD due to increases in hypertension problems. This estimated value may be used by local authorities to set compensation for Yanomami people.

## 4. Discussion

The methodology developed in this paper is the first of its kind to assess the impact on human health caused by mercury used in gold mining. To achieve this methodology, it was necessary to understand the complexity of the mercury cycle and the ASGM processes. First, using this methodology, we presented evidence against the argument that the source of the impacting mercury is deforestation, which releases natural mercury present in the forest [23,24,25,26]. The mobilization of natural mercury by deforestation and forest fires represents a relevant impact [30,31,32,33,34,35,36,37,38,39,40,41,42,43], but the use of mercury by ASGM represents the greatest participation in the release of mercury [44]. In addition, it is important to remember that the artisanal mining activity also causes the mobilization of natural mercury due to the process of excavating the soil and sediment from the rivers [91]. Thus, we can conclude this deforestation contributes to the increase in mercury circulation in the Amazon. However, our focus on this paper is related to impact of mercury released directly by gold mining.

Given that we consider only the additional mercury released from ASGM, another complexity arises from the difficulty in attributing responsibility to specific mines. Such complexity can be explained by the ability of mercury to remain bioavailable for long periods [116], bringing cumulative impacts from ASGM exploration [45,46,72,73,74,75,76,77,78]. Thus, the understanding that there is an increase in methylmercury concentration in the hair of a population [49,50,69,70,71,72,73,123] is not enough information to make a specific gold mine responsible, since this increase in concentration can be explained by the history of exploration of other mines that released mercury into the environment. Therefore, the judicialization process of a specific illegal gold mine becomes more challenging as there are several illegal ASGMs in the Amazon [167].

Given the whole context of mercury and the ASGM, this article proposes an innovative methodology that proposes a linear relationship based on the amount of mercury used by ASGM and its adverse effects on human health. For this, an extensive literature review was performed that tracks the average mercury use of ASGM [17,72,74,87,88], mercury disposal in the environment [17,18,19,20], fish consumption [18,49,50,108,110,111,112,113], and the level of potential contamination harmful to human health [52,53,54,55,56,57,58]. The study is also innovative compared to other studies [10,47,48,49] observed due to the fact that the impacts on human health vary according to the context analysed, such as fish intake in the population and demographic density. This means that a replicable methodology was developed which was adaptable to the different contexts observed within the Amazon

After the study related the use of mercury in gold mining and the average increase in mercury concentration in the population, we explored the literature relating gold extracted and negative effects on human health [72,73,74]. Kahhat et al. [74] used Usetox software, which characterizes chemical impacts on human health and freshwater ecotoxicity and scales an impact of 2 non-cancerous cases and 0.0192 cancerous cases for each kilogram of gold extracted. Similarly, Gulley [72] and Spadaro and Rabl [73] assessed the IQ loss impact for each kilogram of gold from the calibrated benefit transfer approach employed in environmental valuation literature estimates of the impact of mercury on global earnings to twelve gold mining sites around the world. Gulley [72] used studies [37,39,40] that map quantities of mercury emissions into lost earnings due to fetal IQ loss to produce monetary estimates of the impact of mercury emissions. According to Gulley [72], the weighted average estimates an impact of US$ 7300 per kilogram of mercury released into the environment, and this value may increase to the upper limit of US$ 22,300 depending on the assumptions adopted. However, such methodologies are different from the proposal presented in this article, which links mercury use at the beginning of the chain, from the mercury loss in the aquatic environment, to assess the impact on human health. The methodology presented measures the loss of well-being caused using mercury by gold mining. However, other studies [168,169,170,171] value the impact of mercury by the cost of remediation of mercury in vegetation.

We must reinforce that before the development of this methodology, the Brazilian institutions responsible for setting fines for illegal ASGM did not measure the impact on human health from the use of mercury in the mines. This methodological gap is filled with the tool developed in this article, and based on this, institutions such as the Federal Police and Federal Public Prosecution in Brazil have instruments to prosecute illegal gold mining damages in order to stop the advance of this activity in the Amazon.

Nevertheless, this article recognizes the limitations of the relationship between mercury use in gold mining and its impacts on human health. Given the lack of studies that assess the factors that influence the response time between changes in deposition and changes in methylmercury concentrations in fish, in this study we needed to assume the temporal effect of mercury release and fish bioaccumulation over 50 years. In addition, the Amazon region is complex and diverse. The model presented is simplified and does not consider local differences, such as river color and water flow, that could impact the local mercury cycle. Such characteristics need to be further studied.

We did not find any study in Brazil that relates mercury concentration to myocardial infarction. The study by Salonen et al. [8], carried out in Finland, was the only related study found in the literature. Even though both regions have a high intake of fish contaminated by mercury, the physical differences between the populations in the Amazon and in Finland should be further studied for further adaptations.

The limitations presented throughout the article can be overcome with new studies of biophysical mercury dispersion until it reaches humans and possible impacts to human health from increased mercury intake by humans. Long-term monitoring studies that collect mercury concentrations in water, sediments, and fish are needed, particularly in the Amazon. These efforts can lead to long-term data records that can be compared to predictions. Likewise, additional studies should be carried out to assess fish consumption rates in pregnant women, women of childbearing age, and men.

Finally, the methodology presented in this article does not address all impacts on human health since only the outcomes related to the release of mercury in the aquatic environment are measured, not considering the impacts of air exposure due to the inhalation of mercury in the atmosphere (especially in miners) [78,172,173]. The mercury cycle is very complex and, for this reason, the article does not measure the effects caused by the mercury emission into the atmosphere, which can travel long distances. In addition, the article focused on three negative health effects observed in the literature. However, it is possible that there are other health problems associated with exposure to mercury via ingestion of contaminated fish. In this sense, it can be said that the impacts presented are underestimated.

## 5. Conclusions

This article is the first scientific work whose objective was to propose a methodology to quantify the average economic health impact of the extraction of gold by ASGM in Brazil (using the DALY indicator) and convert them into monetary losses (using the VSL indicator).

The application of this methodology to estimate the impacts of mining in Yanomami territory revealed that the 5 km^2^ illegally deforested for ASGM in 2020 would result in severe damages to human health. Based on this, we estimate that around 32 kg of mercury were spilled into rivers, affecting up to 44,000 people. We estimated that 307 people will develop hypertension problems from this activity, 85 people will develop acute myocardial infarction, and 4 children would have born with mild mental retardation. These effects are related only to the presence of small-scale gold mining in 2020, which demonstrates the significant and growing impact with the expansion of this activity in Brazil in recent years. The economic health impact of these health outcomes could reach up to 69 million USD for the 2020 spills alone.

The standardization of mercury impacts assessment is essential for supporting the containment of illegal gold mining activities in the Brazilian Amazon. The developed methodology contributes to the work of control agencies such as the Brazilian Public Prosecutors Office and the Federal Police, who are already using this methodology to estimate fines in the estimation of compensation, showing reliability on the part of these institutions in the scientific results presented. In addition, it contributes to the speed of judicialization in the fight against this illegal activity, and it may support policymakers to plan investments in command-and-control that can prevent the expansion of illegal ASGM.

## Figures and Tables

**Table 1:** Summary of variables, unit of measurement, range values, and source used in this article.

Variables	Unit of Measurement	Range	Source
Distance of mercury dispersion in rivers	kilometers	4–100 km (Dispersal of mercury can be carried out by fish that travel long distances up to 5000 km [131,132,133,134,135,136]. Conservatively, the dispersion of mercury by fish is not considered).	[30,124,125,126,127,128,129,130]
Proportion of mercury used for each kilo of gold extracted	Mercury: Gold	1.6:1–5:1	[17,72,74,87]
Proportion of mercury released into water	Percentage	7%–21%	[17,18,19,20]
Methylation rate in water	Percentage	2%–22%	[91,92,93,94,95,96,97]
Average mercury contamination of fish	Microgram of mercury per gram of fish	0.13–3 (μg Hg/g of fish)	[49,52,53,54,55,56,57,58,104,105,106,107,108,109,110]

**Table 2:** Summary of variables, unit of measurement, range values, and source used in Yanomami case study.

Variables	Unit of Measurement	Value	Source
Area impacted by gold mining in 2020	km^2^	5 km^2^	[164]
Average individual weight of the indigenous population	Kilogram	53.2 kg	[49]
Average daily consumption of fish per rural individual	Grams of fish per person per day	100 g/person/day (Average between indigenous (100 g/person/day) [49] and Riverside (189 g/person/day) [50])	[49]
Average population density	Inhabitants by km^2^	2 inhab/km^2^	[156]
Urban population (state of Roraima as a whole)	Percentage	76%	[156]
Rural population (state of Roraima as a whole)	Percentage	24%	[156]
Distance of mercury dispersion in rivers	kilometers	100 km	[116]
Proportion of mercury used for each kilo of gold extracted	Mercury: Gold	2.6:1	[17]
Proportion of mercury released into water	Percentage	13%	[20]
Methylation rate in water	Percentage	2%	[91]
Average mercury contamination of fish	Microgram of mercury per gram of fish	0.5 (μg Hg/g of fish)	[110]

## Data Availability

Data Availability in http://calculadora.conservation-strategy.org/#/ (accessed on 4 November 2021).
